# Successful treatment of a *Candida auris* intra-articular infection

**DOI:** 10.1080/22221751.2019.1625287

**Published:** 2019-06-09

**Authors:** Scott C. Roberts, Teresa R. Zembower, Maureen K. Bolon, Anish R. Kadakia, Jasen H. Gilley, Jason H. Ko, Jessica Clark, Sharon Ward-Fore, Babafemi O. Taiwo

**Affiliations:** a Division of Infectious Diseases, Northwestern University Feinberg School of Medicine, Chicago, IL, USA; b Department of Pathology, Northwestern University Feinberg School of Medicine, Chicago, IL, USA; c Division of Orthopaedic Surgery, Northwestern University Feinberg School of Medicine, Chicago, IL, USA; d Division of Plastic Surgery, Northwestern University Feinberg School of Medicine, Chicago, IL, USA

**Keywords:** *Candida auris*, amphotericin cement spacer, intra-articular infection

## Abstract

A 78-year-old woman with a long-term ankle spacer with antibacterials developed an intra-articular *Candida auris* infection. Treatment with systemic antifungal therapy plus an amphotericin B moulded cement spacer was successful.

## Case

A 78-year-old female Chicagoland native with a history of a left ankle fracture treated with open reduction and internal fixation in 1990 presented to her local orthopedic surgeon with chronic pain and underwent a left total ankle replacement in November 2016. She did well post-operatively until approximately one month later when she developed wound dehiscence. Superficial wound cultures were negative and after progression on 18 days of amoxicillin/clavulanic acid (875–125 mg) twice-daily, she underwent removal of the hardware, placement of a Palacos^®^ (Heraeus Medical, Hanau, Germany) cement spacer mixed with 1 g of vancomycin and 1.2 g of tobramycin per 40 g of cement, anterior tibial tendon and extensor hallucis longus tendon removal, and left free vastus lateralis flap with a split-thickness skin graft to the open wound site. Operative cultures were negative and she received 6 weeks of empiric intravenous vancomycin 1 g every 8 h and ceftriaxone 2 g every 24 h, then oral ciprofloxacin 500 mg twice-daily and sulfamethoxazole/trimethoprim 800–160 mg twice-daily for an additional three weeks. The wound closed around March 2017; however, 15 months later in June 2018 she presented to plastic surgery clinic with a 1 cm wound dehiscence. She was otherwise well, living at home, and ambulating without any recent travel or hospital exposures except for wound care and outpatient follow up appointments at her local facility. She underwent a deep wound debridement, closure with flap advancement and allograft superficial peroneal nerve reconstruction. Operative findings included fibrinous tissue with the appearance of biofilm. The spacer was not removed as it did not appear involved. Deep wound cultures obtained from this procedure were plated on Blood, Chocolate and MacConkey agar and grew *Candida auris* in pure culture in moderate amounts after 2 days on Blood and MacConkey agar plates*.* Identification was confirmed via 18S rRNA sequencing using the ITS1/ITS4 primer sets and D1/D2 DNA sequencing along with combined phenotypic characterization. *Candida auris* identification was obtained through matching ITS sequences with previously established signatures in the GenBank database. Antifungal susceptibility testing was performed using Sensititre™ YeastOne™ YO3IVD AST Plate (Thermo Scientific™, West Sussex, UK) and minimum inhibitory concentration (MIC) values (in µg/mL) were: fluconazole: 2, micafungin: 0.06, caspofungin: 0.125, 5-fluorocytosine: <0.125, itraconazole: 0.125, voriconazole: <0.03, and amphotericin B: 0.5. The patient was started on oral fluconazole 400 mg daily. Two weeks later, her wound showed increasing erythema and a new area of purulent drainage. Daily fluconazole dose was increased to 600 mg, and she was admitted for surgical intervention. On admission, she was afebrile and hemodynamically stable. A complete blood count and a comprehensive metabolic panel were normal. Given a lack of systemic symptoms, blood cultures were not obtained. She underwent antibiotic spacer removal, debridement of the remaining extensor tendons, partial excision of necrotic distal tibia and talus bones, and placement of a moulded spacer composed of 40 g Palacos^®^ cement mixed with 100 mg of heat-stable powdered amphotericin B deoxycholate (Fungizone). Operative cultures were negative. Postoperatively, she received intravenous micafungin 100 mg daily for 2 weeks followed by oral fluconazole 400 mg daily. The wound has healed and she has regained mobility without any assistive device. She recently noticed onset of hair loss, presumably due to prolonged use of fluconazole. The patient has completed six months of fluconazole 400 mg daily with plans to continue this for at least a year followed by a lower dose, if tolerated.

## Discussion

*C. auris* is an emerging nosocomial pathogen with a high potential for multidrug-resistance. First isolated from the external ear canal of a patient in 2009, *C. auris* has now been detected in 6 continents and 32 countries as of 2018 [1,2]. Prevalence is likely underestimated since *C. auris* is prone to misidentification, as there are several phylogenetically similar strains such as *Candida haemulonii*, *Candida famata, Candida sake, Rhodotorula glutinis*, and *Saccharomyces cerevisiae* [3,4]. *Candida auris* strains are clonal and transmissible, and genomic analyses have shown United States isolates are derived from four global regions spread through international travel [5,6].

Our case is noteworthy because bone and joint involvement of *C. auris* has been rarely reported, and no detailed cases describing spacer and joint involvement exist. Further, antifungal spacer placement, while sometimes utilized for other *Candida* species, has yet to be described for *C. auris* [7,8]. Notably, antifungal breakpoints are tentative, and optimal treatment of orthopedic *C. auris* infection remains speculative with only a few case reports and expert opinions available to assist clinicians. One case describes chronic otomastoiditis that was successfully treated with fluconazole [9]. Another notes sternal osteomyelitis treated with posaconazole, although the patient died shortly afterwards from an unrelated cause [10]. The treatments offered to both cases were based on in-vitro MIC results, as with our case; however, at least one study has found recurrent fluconazole-sensitive *C. auris* detection from the urine in spite of fluconazole therapy [11]. A third study detected *C. auris* in the femur of a patient who improved on fluconazole therapy despite an MIC of 256 µg/mL, likely reflecting colonization [12]. Few other studies have reported *C. auris* isolation in bone and joint spaces although details are unclear. Here, we describe the treatment of a fluconazole-sensitive *C. auris* intra-articular infection. Our patient received surgical debridement and placement of an amphotericin-impregnated spacer in addition to systemic antifungal therapy, primarily with fluconazole plus two weeks of micafungin given immediately post-surgery.

A cement spacer mixed with powdered amphotericin B was placed given its heat-stable properties, powdered formulations, and the mildly elevated MIC to fluconazole, although the MIC was below the tentative susceptibility cut-off for *C. auris* [13]. Amphotericin B-impregnated spacers have been utilized for prior *Candida* infections, but not *C. auris*. Most, but not all, report eradication of infection when used in conjunction with systemic antifungal therapy. However, numerous challenges exist in using antifungal spacers. Doses in literature are variable and local bone and joint space antifungal concentrations are unknown. Additionally, certain cement mixtures may impact effectiveness; one study found mixture with polymethyl methacrylate, a common component of spacers, increased compressive strength of the cement, however local amphotericin elution was decreased [14]. Concern also exists for the development of resistance given local antifungal dilution, and systemic absorption of amphotericin B may provoke nephrotoxicity.

Our patient has developed alopecia, an uncommon adverse effect of fluconazole seen in doses of 400 mg daily for at least two months and reversible upon discontinuation [15]. This may affect her willingness to take fluconazole long-term.

Infection prevention and control procedures for *C. auris* have yet to be optimized. Nosocomial outbreaks have been described, and patients can be colonized for months [16–18]. Current recommendations include Standard and Contact Precautions while colonized with reassessments for colonization every 3 months through axillary and groin swabs as well as the prior site of isolation [13]. Recommended strategies to prevent nosocomial spread of *C. auris* in the operative setting include routine precautions for prevention of surgical site infections, which were employed in this case [13]. Preoperative infusion of a single dose of micafungin was implemented to control a recent outbreak of largely fluconazole-resistant *C. auris* in the United Kingdom [16]; this was not performed in our patient. Further precautions applied at our facility included education of the operating room, inpatient, and outpatient staff regarding the organism and required infection prevention measures, surgical space suits and shoe covers worn by all operating room staff, bleach and UV light technology cleaning of the operating room, preoperative, and postoperative care units, contact precautions on the inpatient wards with gowns and gloves, 1:1 nursing care, daily room cleanings with bleach containing solution, limited shared equipment that was disposable when able, and prohibited hallway walking. Upon discharge the room was terminally cleaned, disposable curtains changed, and the room was cleaned using UV light technology. For outpatient care, the patient was scheduled as the last visit of the day, was transferred from check-in directly to the room, and was instructed to keep all wounds covered until examination. Additionally, follow-up appointments with orthopedics and infectious diseases were combined to one location and staff wore gloves and gowns with outpatient rooms cleaned with bleach containing solution followed by UV light technology.

This case highlights a unique case of *C. auris* infection and our management approach. In contrast to previously reports of azole resistance rates of up to 90%, our isolate was presumed fluconazole sensitive with an MIC of 2 [19]. As this was the first case of *C. auris* seen at our hospital and no cases were present concurrently, acquired mechanisms of resistance in outbreak settings, such as Y132F and K143R amino acid substitutions in the ERG11 gene, were likely not present [19]. Given a known predisposition for the development of azole resistance with prolonged therapy, systemic echinocandin therapy and an amphotericin B-impregnated spacer were used concurrently. Further insight is needed to determine the utility of the amphotericin B-impregnated spacer used in our patient. We will continue to monitor her since late relapse of intra-articular infection can occur after apparently successful treatment.
